# Effect of MRI acquisition parameters on accuracy and precision of phase-contrast measurements in a small-lumen vessel phantom

**DOI:** 10.1186/s41747-024-00435-3

**Published:** 2024-03-13

**Authors:** Maria Correia de Verdier, Johan Berglund, Johan Wikström

**Affiliations:** 1 Department of Surgical Sciences, Section of Neuroradiology, Uppsala University, Uppsala, Sweden; 2 Department of Surgical Sciences, Section of Molecular Imaging and Medical Physics, Uppsala University, Uppsala, Sweden

**Keywords:** Blood vessels, Blood flow velocity, Magnetic resonance angiography, Magnetic resonance imaging, Phantom studies

## Abstract

**Background:**

Phase-contrast magnetic resonance imaging (PC-MRI) quantifies blood flow and velocity noninvasively. Challenges arise in neurovascular disorders due to small vessels. We evaluated the impact of voxel size, number of signal averages (NSA), and velocity encoding (VENC) on PC-MRI measurement accuracy and precision in a small-lumen vessel phantom.

**Methods:**

We constructed an *in vitro* model with a constant flow rate using a 2.2-mm inner diameter plastic tube. A reservoir with a weight scale and timer was used as standard reference. Gradient-echo T1 weighted PC-MRI sequence was performed on a 3-T scanner with varying voxel size (2.5, 5.0, 7.5 mm^3^), NSA (1, 2, 3), and VENC (200, 300, 400 cm/s). We repeated measurements nine times per setting, calculating mean flow rate, maximum velocity, and least detectable difference (LDD).

**Results:**

PC-MRI flow measurements were higher than standard reference values (mean ranging from 7.3 to 9.5 mL/s compared with 6.6 mL/s). Decreased voxel size improved accuracy, reducing flow rate measurements from 9.5 to 7.3 mL/s. The LDD for flow rate and velocity varied between 1 and 5%. The LDD for flow rate decreased with increased voxel size and NSA (*p* = 0.033 and 0.042). The LDD for velocity decreased with increased voxel size (*p* < 10^-16^). No change was observed when VENC varied.

**Conclusions:**

PC-MRI overestimated flow. However, it has high precision in a small-vessel phantom with constant flow rate. Improved accuracy was obtained with increasing spatial resolution (smaller voxels). Improved precision was obtained with increasing signal-to-noise ratio (larger voxels and/or higher NSA).

**Relevance statement:**

Phase-contrast MRI is clinically used in large vessels. To further investigate the possibility of using phase-contrast MRI for smaller intracranial vessels in neurovascular disorders, we need to understand how acquisition parameters affect phase-contrast MRI-measured flow rate and velocity in small vessels.

**Key points:**

• PC-MRI measures flow and velocity in a small lumen phantom with high precision but overestimates flow rate.

• The precision of PC-MRI measurements matches the precision of standard reference for flow rate measurements.

• Optimizing PC-MRI settings can enhance accuracy and precision in flow rate and velocity measurements.

**Graphical Abstract:**

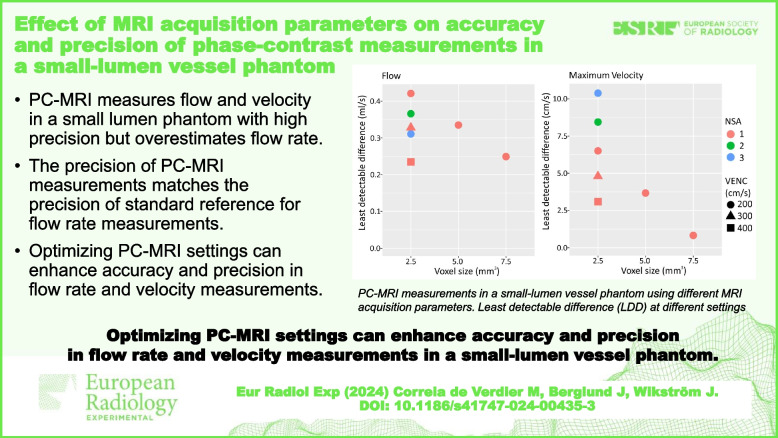

## Background

Phase-contrast MRI (PC-MRI) is a noninvasive technique to quantify blood flow and velocity in the cardiovascular system [1]. This technique is used for measuring blood flow in a range of clinical applications, most commonly for assessment within the heart and great vessels, but measurement in small arteries such as intracranial, renal, and coronary arteries is also possible [2–6].

When performing flow and velocity measurements in the vascular system, both accuracy and precision are important properties. Variations in acquisition parameters, including voxel size, number of signal averages (NSA), and velocity encoding (VENC), have been shown to influence both the accuracy and precision of PC-MRI measurement [2, 7–9].

The use of PC-MRI in neurovascular diseases is especially challenging because the vessel lumen mean diameter in the anterior, middle, and posterior cerebral arteries is only 1–3 mm [10], which is smaller than the lumen used in most previous flow phantom studies. Previous studies of pulsatile flow on vessel phantom with a diameter of 2 mm on a 1.5-T scanner, found underestimation of flow rate and increased flow rate values with increased voxel size, but no assessment was made on the effect of NSA or VENC variations [11, 12].

To be able to further investigate the possibility of using PC-MRI for neurovascular disorders, we need to gain more knowledge on the influence of acquisition parameters on PC-MRI-measured flow and velocity in small caliber vessels. Therefore, in this study, we assessed the effect of spatial resolution, NSA, and VENC on the accuracy and precision of PC-MRI measurements at 3 T in a vessel phantom with small lumen diameter. We also determined the optimal setting for these parameters to examine a small-lumen vessel.

## Methods

### Data acquisition

**Fig. 1:**
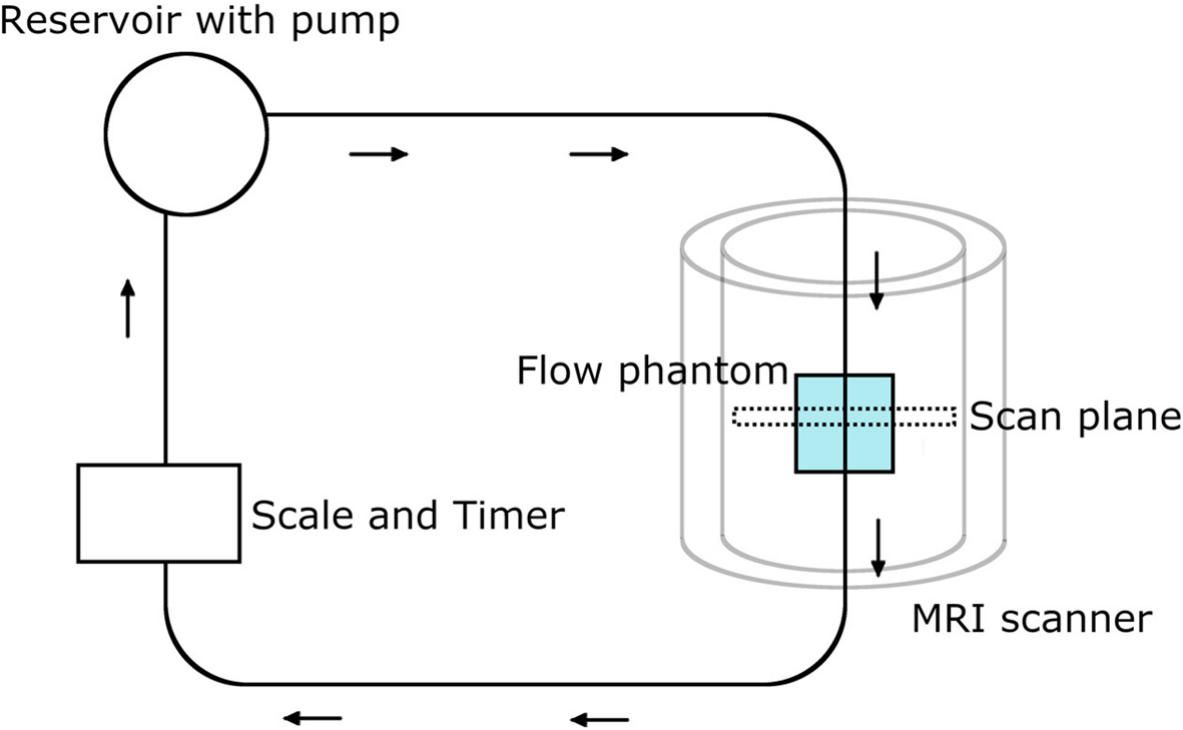
Schematic drawing of the flow model. A 10-L water reservoir guaranteed a constant preload to the pump. The flow model comprised a submersible pump system and plastic tubes. The arrows indicate the flow direction. The flow phantom consisted of a plastic tube with an inner diameter of 2.2 mm passing through a plastic box filled with agar gel. The phantom was positioned inside the head coil in the scanner with the direction of flow parallel to the long axis of the bore and the scan plane perpendicular to the plastic tube in the flow phantom. The flow rate was controlled with a reservoir with a weight scale and timer placed at one end of the flow model and measured at eight different times during the entire experiment

A 3-T scanner (Achieva 3 T X, Philips Medical Systems, Best, The Netherlands) and a 32-channel head coil were used for all the PC-MRI measurements. An *in vitro* flow model was constructed to provide a constant flow rate (Fig. 1). The phantom consisted of a plastic tube with inner diameter of 2.2 mm passing through a plastic box filled with agar gel. A 10-L water reservoir guaranteed a constant preload to the pump. Four mM of copper (II) sulfate pentahydrate was added to the water to reduce T1 relaxation time for improved signal, resulting in a T1 relaxation time of 422 ms at room temperature. These methods were adopted because the differences being studied were potentially very small. The flow rate was controlled with a reservoir with a weight scale and timer placed at one end of the flow model. Pump stability was monitored by measuring flow rate at eight different times during the entire experiment, and the mean value was used as standard reference for flow. No standard reference was used for velocity.

A gradient-echo T1-weighted PC-MRI sequence was performed. The following PC-MRI parameters were used as the baseline setting: repetition time/echo time 20/8 ms, flip angle 15°, bandwidth 217 Hz/pixel, acquired voxel size 0.59/0.84/5 mm^3^, NSA 1 and VENC 200 cm/s. As the aim of this study was to determine the optimum settings for small vessels, we used retrospective gating (artificial gating device) with 70 beats per minute and 12 phases per cardiac cycle to allow acquisition at multiple time points throughout the cardiac cycle *in vivo*. The acquisition time for the baseline setting was 3:28 min:s. Voxel size, NSA, and VENC were changed according to the experiment protocol shown in Table 1. The parameters and range of values in the experiment protocol were chosen because they would be adaptable to clinical PC-MRI measurements in small arteries in patients. We examined three different settings for each parameter, changing one parameter at a time. We also examined the effect of spatial resolution and the highest possible signal-to-noise ratio (SNR), changing voxel size combined with the highest NSA and lowest VENC. A power analysis was performed in order to determine the number of repetitions required with the available time at the MRI scanner. Based on the result, nine different settings were used, and the measurements at each setting were repeated nine times without phantom repositioning. All images were interpolated to a reconstructed pixel size of 0.59 × 0.59 mm^2^.

**Table 1:** PC-MRI acquisition parameters

Parameters	Baseline setting	Test range
Repetition time (ms)	20	Constant
Echo time (ms)	8	Constant
Flip angle (degrees)	15	Constant
Bandwidth (Hz/pixel)	217	Constant
Heart phases	12	Constant
Slice thickness (mm)	5	Constant
Voxel size (mm^3^)	2.5	2.5, 5.0, 7.5
Number of signal averages	1	1, 2, 3
Velocity encoding (cm/s)	200	200, 300, 400

*PC-MRI* Phase-contrast magnetic resonance imaging

### Data analysis

**Fig. 2:**
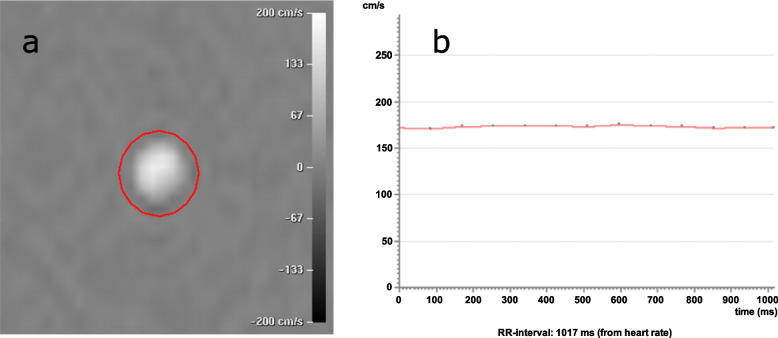
Measurements at the baseline setting. Phase-difference image obtained in one of the 12 phases during the cardiac cycle (**a**). A region of interest surrounds the plastic tube in the flow phantom. Curve representing maximum velocity obtained in all 12 phases (**b**)

PC-MRI data were analyzed using commercially available software (Extended MR WorkSpace 2.6.3.5, Philips Medical Systems, Best, The Netherlands). An elliptic region of interest (ROI) of 17 mm^2^ (50 pixels) was drawn by one of the authors (M.C.V.) around the vessel in the phase-difference image (Fig. 2). ROI size was chosen based on the signal properties of phase-difference images at the baseline setting. Flow rate and maximum velocity were calculated for each of the 12 phases during each repetition, with flow rate defined as mean flow rate over the ROI and maximum velocity defined as the highest pixel value over the ROI.

The mean values for flow rate and maximum velocity were calculated for each repetition. The accuracy of our phantom was determined by comparing PC-MRI flow rate measurements at the baseline setting with measurements in our standard reference. To illustrate the effect of the acquisition parameters on flow rate velocity measurements, graphs of the velocity across the flow phantom lumen were drawn.

### Statistical analysis

Statistical analysis was performed using R, version 4.1.2, 2021 (R Foundation for Statistical Computing, Vienna, Austria) [13]. To assess accuracy, we calculated the relative error for PC-MRI flow rate as the difference between the PC-MRI flow measurement and the standard reference, divided by the standard reference. To assess precision, two methods were used.

1. The coefficient of variation (CoV) was calculated as the ratio of the standard deviation (SD) to the mean, multiplied by 100 to express it as a percentage. Where the SD and mean of each setting were used.

2. The reproducibility coefficient was calculated as 1.96 times the SD. The reproducibility coefficient was used to represent the least detectable difference (LDD). The LDD was calculated for both absolute and relative values (given in % of the mean). Breusch-Pagan tests were used to test for differences in the LDD between different settings [14]. The alternative hypothesis was that the LDD changed in a pre-specified direction when the settings were increased (increased voxel size gives lower LDD, increased NSA gives lower LDD and increased VENC gives higher LDD), and consequently, one-sided tests were used.

The *p*-values were computed using the permutation distribution of the Breusch-Pagan statistic and adjusted for multiplicity using the Holm-Bonferroni procedure (adjusted *p*-values are reported). Values of *p* < 0.05 were considered statistically significant. To illustrate the results of the Breusch-Pagan test, a graph was made using LDD values calculated from the regression model that combined all measurements.

## Results

The flow rate measurement in our standard reference was 6.59 mL/s. One of the measurements was considered an outlier and excluded from the analysis (range with outlier 6.25–6.68 mL/s, mean 6.55, median 6.58 and range without outlier 6.50–6.68 mL/s, mean 6.59, median 6.58).

**Fig. 3:**
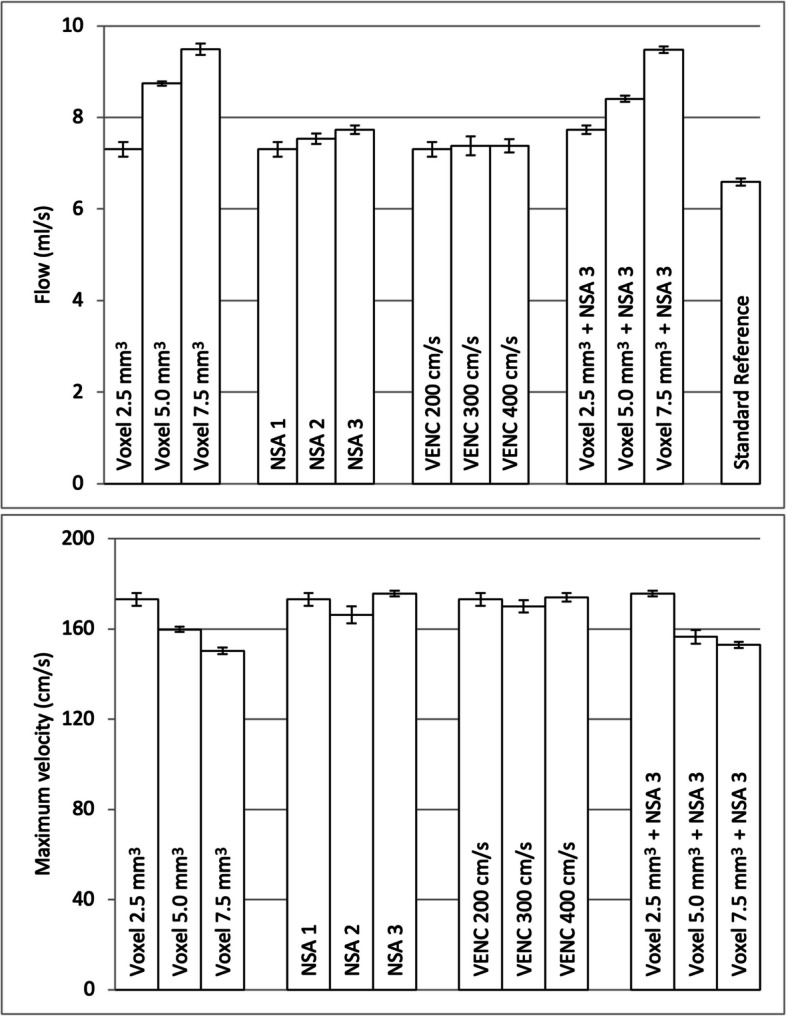
PC-MRI measurements in a small-lumen vessel phantom with different MRI acquisition parameters. Mean and standard deviation of flow rate and maximum velocity measured with PC-MRI and standard reference for flow rate. *PC-MRI* Phase-contrast magnetic resonance imaging, *NSA* Number of signal averages, *VENC* Velocity encoding

All PC-MRI flow rate measurements were higher than our standard reference (mean values ranging from 7.3 to 9.5 mL/s compared with 6.6 mL/s), see Fig. 3. The measured flow rate at our baseline setting was 11% higher than our standard reference. Flow rate measurements increased and velocity measurements decreased with increased voxel size (Fig. 3).

**Fig. 4:**
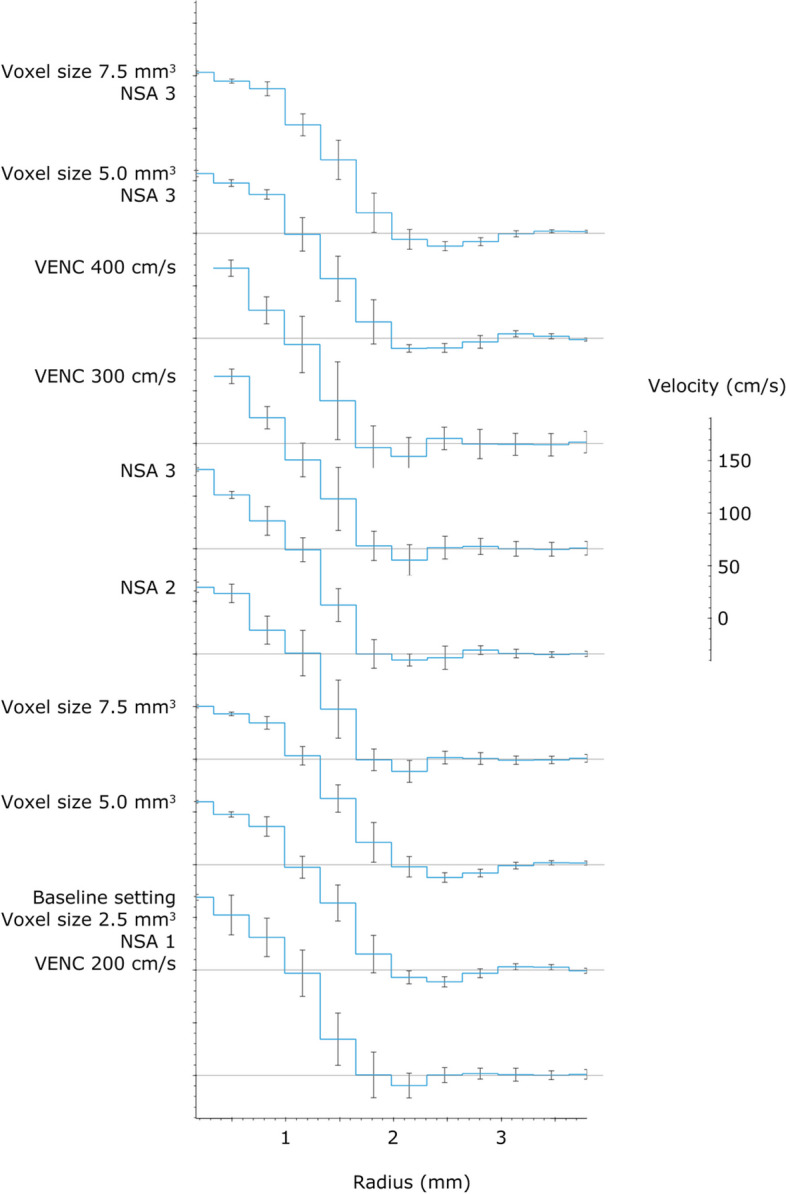
PC-MRI measurements in vessel phantom with inner diameter 2.2 mm and with different acquisition parameters. The flow rate velocities were binned according to the radial distance from the tube center. The plot shows the mean and standard deviation calculated over all phases and repetitions. The velocity scale for individual acquisition parameters is shown separately. Gray horizontal lines represent 0 cm/s for each acquisition parameter, with the first gray line at the bottom pertaining to the baseline setting. *PC-MRI* Phase-contrast magnetic resonance imaging, *NSA* Number of signal averages, *VENC* Velocity encoding

Graphs of the velocity across the flow phantom lumen are illustrated in Fig. 4. The LDD for flow rate was 0.32 mL/s for PC-MRI at baseline setting and 0.15 mL/s for the standard reference. Results from all the measurements are shown in Tables 2 and 3.

**Table 2:** Results from PC-MRI-measured flow rate with different acquisition parameters

Protocol			Flow rate (mean and range, mL/s)	SD	CoV (%)	LDD (mL/s)
Voxel (mm^3^)	NSA	VENC (mL/s)				
2.5	1	200	7.30 (7.05–7.61)	0.16	2	0.32 (4%)
5.0	1	200	8.74 (8.67–8.83)	0.05	1	0.10 (1%)
7.5	1	200	9.49 (9.20–9.59)	0.12	1	0.24 (3%)
2.5	2	200	7.54 (7.36–7.71)	0.12	2	0.23 (3%)
2.5	3	200	7.73 (7.58–7.90)	0.09	1	0.18 (2%)
2.5	1	300	7.38 (7.08–7.70)	0.21	3	0.41 (5%)
2.5	1	400	7.38 (7.04–7.52)	0.14	2	0.28 (4%)
5.0	3	200	8.41 (8.33–8.50)	0.07	1	0.13 (2%)
7.5	3	200	9.48 (9.33–9.58)	0.08	1	0.15 (2%)
Reference standard			6.59 (6.50–6–70)	0.08	1	0.15 (2%)

*CoV* Coefficient of variation, *LDD* Least detectable difference, in both absolute and in relative values (given as % of the mean), *NSA* Number of signal averages, *PC-MRI* Phase-contrast magnetic resonance imaging, *SD* Standard deviation, *VENC* Velocity encoding

**Table 3:** Results of PC-MRI measured maximum velocity with different acquisition parameters

Protocol			Maximum velocity (mean and range, cm/s)	SD	CoV (%)	LDD (cm/s)
Voxel (mm^3^)	NSA	VENC (mL/s)				
2.5	1	200	173 (168–177)	2.86	2	5.60 (3%)
5.0	1	200	160 (158–162)	1.13	1	2.21 (1%)
7.5	1	200	150 (149–154)	1.45	1	2.84 (2%)
2.5	2	200	166 (161–174)	3.82	2	7.49 (5%)
2.5	3	200	176 (174–178)	1.23	1	2.42 (1%)
2.5	1	300	170 (164–173)	2.78	2	5.45 (3%)
2.5	1	400	174 (172–177)	1.94	1	3.80 (2%)
5.0	3	200	156 (153–161)	3.12	2	6.12 (4%)
7.5	3	200	153 (151–155)	1.34	1	2.62 (2%)

*CoV* Coefficient of variation, *NSA* Number of signal averages, *LDD* Least detectable difference, in both absolute and in relative values (given as % of the mean), *PC-MRI* Phase-contrast magnetic resonance imaging, *SD* Standard deviation, *VENC* Velocity encoding

**Fig. 5:**
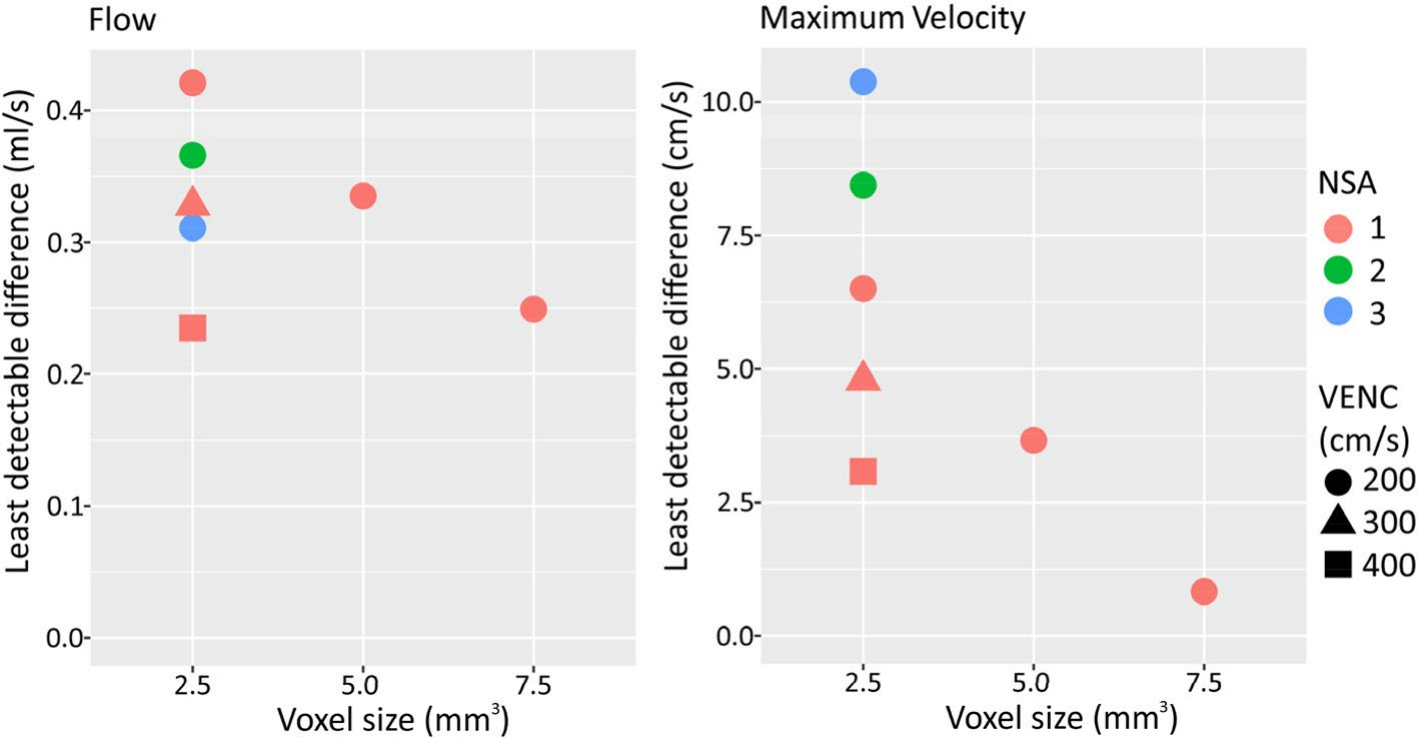
PC-MRI measurements in a small-lumen vessel phantom using different acquisition parameters. Least detectable difference at different settings, based on a regression model that combined all measurements. *PC-MRI* Phase-contrast magnetic resonance imaging, *NSA* Number of signal averages, *VENC* Velocity encoding

We found a decrease in the LDD for flow rate with increasing voxel size (*p* = 0.033) and NSA (*p* = 0.042), but no change when VENC was increased (*p* = 1.000, Fig. 5). We found a decrease in the LDD for maximum velocity when voxel size was increased (*p* < 10^-16^), but no change when NSA or VENC was increased (*p* = 1.000, Fig. 5).

## Discussion

In this study, we examined the accuracy and precision of PC-MRI measurements in a vessel phantom with small lumen diameter and constant flow rate. We also explored the impact of change in spatial resolution, NSA, and VENC on PC-MRI measurements. We found that flow measured with PC-MRI was higher than that in our standard reference. Improved accuracy was obtained with decreased voxel size. We found good precision in flow rate measurements with PC-MRI; at the baseline setting the precision was similar to the precision in our standard reference. Improved precision for both flow rate and velocity measurements was obtained with increased voxel size. Improved precision for flow rate was also observed with increased NSA.

### Challenges in measuring flow rate in small vessels

In PC-MRI, the intensity of each voxel in the phase images is proportional to the mean velocity within the volume element. When examining vessels with a small lumen, inaccurate measurements due to variation of phase and signal amplitude within a voxel will occur (*e.g.*, partial volume effects at the vessel boundary and intraluminal intravoxel phase dispersion in laminar flow) [7, 9, 15, 16]. In laminar flow, slower inflowing spins closer to the vessel wall will experience more radiofrequency pulses than faster spins closer to the vessel center. As a result, the signal from faster inflowing spins will be less saturated, leading to a stronger signal in the center (inflow effect) [17].

### Partial volume effects

In boundary voxels (which comprise both stationary and moving spins), the voxel value will be the moving spins velocity integrated over the whole voxel volume, resulting in partial volume effects leading to velocity underestimation and vessel area overestimation [7, 18]. If the signal magnitude is the same in the vessel lumen compared to the vessel wall and surrounding tissue, these two counteracting effects will cancel each other out, and the measured flow rate will be correct. Higher signal in the vessel lumen due to inflow effects and differences in relaxation time in different tissues will lead to a larger contribution from the vessel lumen compared to the vessel wall and surrounding tissue, resulting in overestimation of the mean velocity in the voxel.

### Intravoxel phase dispersion

In laminar flow, voxels comprising only moving spins will experience intraluminal intravoxel phase dispersion due to spins with different velocities within those voxels. Due to the inflow effect and stronger signal from faster inflowing spins with higher velocity, the mean velocity within a voxel will be overestimated, and as the vessel area remains the same, the flow rate will also be overestimated.

### Accuracy and precision at baseline setting

In our study, the flow rate measured with PC-MRI was higher than our standard reference. Greater accuracy has been found in studies on larger vessels [12, 19–21]. We believe that the overestimation seen in our study was caused by a larger contribution of both partial volume effects at the vessel boundary and intraluminal intravoxel phase dispersion when small vessels with limited spatial resolution are examined. Other studies have shown an underestimation in flow rate when small vessels were examined, but these studies were performed in a phantom with pulsatile flow [11, 12]. In pulsatile flow that resembles arterial pulsation with a shorter systolic than diastolic phase, inadequate temporal resolution is also a source of inaccuracy, and as maximum velocity is underestimated, the flow rate will also be underestimated. Precision was overall good for PC-MRI measurements, but precision was poorer at baseline setting than for our standard reference.

### Voxel size and spatial resolution

We found an increased overestimation of flow rate with increased voxel size. Higher flow rate values with decreasing spatial resolution have also been observed in previous studies [2, 7, 9, 11, 15]. With increased voxel size, the proportion of partially occupied voxels at the vessel boundary and different velocities within an intraluminal voxel will increase, and as a result, the influence from partial volume effects and intravoxel phase dispersion will be larger. As a consequence, increased voxel size will yield increased overestimation of flow rate. Another aspect with decreased spatial resolution is the truncation artifact, which arises at the interphase between flowing and stationary matter in the vessel wall. This will cause the background phase signal to diverge from zero, see Fig. 4. With increased voxel size the shape of the curve in Fig. 4 changes. The truncation artifact increases as the spatial resolution decreases, with increased overestimation of flow rate with increased voxel size [22]. As an effect, flow rate measurement will greatly depend on ROI size. With increased voxel size we found a decrease in the maximum velocity, which was also observed by Lotz et al. in an experiment with a 15-mm phantom with constant flow rate [2]. As previously mentioned, different velocities are found within one voxel in laminar flow, which causes intravoxel phase dispersion, and an average velocity rather than the maximum velocity is measured.

### Voxel size and signal-to-noise ratio

The SNR will also affect flow velocity measurements, and small differences in velocity and slow flow might be missed if SNR is too low. Both parameters influencing the amount of noise in the image and signal from flowing spins will influence SNR. Voxel size will, apart from spatial resolution, also influence SNR. This is due to the fact that the signal in the image is dependent on both voxel size and total sampling time. Sampling time will be determined by the NSA, samples of the phase encoding direction, samples of the frequency encoding direction, and bandwidth. SNR is directly proportional to voxel size if sampling time is held constant, and increased voxel size will result in higher SNR and decreased random error. In our study, increasing voxel size yielded increased precision regarding both flow and velocity. Wolf et al. have also shown increased precision in flow rate measurements with increased voxel size [16].

### Number of signal averages

In our study, no change in accuracy was observed when NSA was altered. Increasing NSA improved precision regarding flow rate, but we could not see any change in precision regarding maximum velocity. Bakker et al. performed PC-MRI measurements on a flow phantom with an inner diameter of 5.3 mm using pulsatile flow and also found increased precision of flow rate measurements when NSA was increased stepwise from 1 to 16 [8]. As increasing NSA will increase imaging time, we chose to use values that could be applied in a clinical setting in our experiment protocol. One possible explanation for the finding of improved precision regarding flow rate, but not maximum velocity, is that the true variation in maximum velocity has the same order of magnitude as the random error. Another possible explanation is that there might be small movements in the phantom during the measurements (due to the gradients) and that maximum velocity is more sensitive to the placement of the voxels than is flow. When changing voxel size, we found improvement in both flow rate and maximum velocity, but in our experiment protocol, the steps between different voxel sizes had a larger impact on SNR than the different NSA settings because the SNR is directly proportional to voxel size, but to the square root of the NSA.

### Velocity encoding

Another parameter with possible influence on PC-MRI measurements is VENC. If the VENC is set to high, the SNR of the measured velocity will decrease [2, 23, 24]. In our study, no change in accuracy or precision was observed when VENC was increased. Theoretically, increased VENC (with decreased SNR) would be expected to affect the precision of maximum velocity but have less influence on flow rate. Since flow is the averaged velocity for a several voxels, noise will have a smaller impact on the measurement. Previous studies on both pulsatile and constant flow have shown that an increase in VENC causes a decrease in precision in flow rate and velocity measurements with increased overestimation of maximum velocity [2, 8, 24]. Other studies have found no change in accuracy or precision of PC-MRI flow rate measurements with changing VENC [9, 25]. We believe that the reason for the difference in results is that the VENC setting in our study was not altered enough to change the outcome. It is possible to compensate for larger VENC by improving SNR with larger voxel size, increased imaging time, or imaging at higher field strength. We used a 3-T scanner in which the tolerance for the choice of VENC is larger compared to a 1.5-T scanner [19]. A larger VENC setting is also tolerated if a gated PC-MRI sequence is used rather than a non-gated sequence [25].

### *PC-MRI measurements in intracranial vessels *in vivo

It is difficult to assess the accuracy of PC-MRI measurements *in vivo* because there is no reference standard. Blood velocity can be assessed in intracranial vessels with transcranial Doppler ultrasound. Doppler ultrasound is widely used but has the disadvantage of overestimating maximum velocity [26]. Other disadvantages of transcranial Doppler ultrasound are that it is an operator-dependent technique, that there is potential lack of acoustic window in the skull, and that measurement errors can be caused by misalignment between the ultrasound beam and flow direction in the vessel. In previous studies comparing ultrasound and PC-MRI in intracranial vessels, PC-MRI has shown lower results for maximum velocity [27–29]. As discussed above, this might be caused by overestimation of the maximum velocity with ultrasound but also that the actual peak velocity in a pulsatile flow is underestimated in cardiac-gated PC-MRI because of limited temporal resolution and averaging over several cardiac cycles. In other studies examining intracranial arteries with PC-MRI, reproducibility is lower than in our study [4, 30]. The difference in results is probably due to different study conditions (phantom with constant flow *versus* pulsatile flow and physiological variations *in vivo*).

### Optimal setting

PC-MRI covers a wide range of clinical applications, and the derived measurements could allow differentiation between pathological and normal blood flow rate. PC-MRI has been used to examine patients with intracranial arteriovenous malformations, where flow rate has been found to be increased in feeding arteries by more than 50% compared to the contralateral side and decreased by approximately 40% after treatment [31, 32]. The reason for using this non-invasive technique to gain hemodynamic information in patients with neurovascular diseases is to increase our knowledge and improve patient care. It is therefore important to optimize MRI acquisition parameters to be able to detect more subtle changes in patients with other neurovascular disorders (*e.g.*, atherosclerosis, vasculitis, Moya-Moya disease) When selecting the optimal setting for PC-MRI examination, you must first determine the measurement task, choosing whether the main goal is to achieve optimal accuracy or precision. If the goal is optimal accuracy (*e.g.*, diagnosing a disease), then high spatial resolution is required. If precision in repeated measurements is desirable (*e.g.*, monitoring treatment response), high SNR is preferred (*e.g.*, larger voxel size, increasing NSA). In a clinical setting, we need to achieve a balance between accuracy and precision. Thus, it is not advisable to measure with high precision if we know the value will be highly inaccurate. In practice, a result cannot be considered highly accurate if the precision of the method is low.

Maximum velocity is less influenced than flow rate by artifacts resulting from changes in spatial resolution (*e.g.*, partial volume artifacts at the vessel wall and truncation artifacts). If the purpose of PC-MRI measurements is to assess hemodynamic changes in a patient (in an artery with pulsatile flow), the diastolic velocity is likely the most reliable parameter because the velocity in the diastolic phase is less variable and hence not as affected by temporal resolution as in the systolic phase. In our study, neither NSA nor VENC influenced the LDD for maximum velocity.

When performing PC-MRI *in vivo* another aspect to consider is imaging time, both time for each sequence but also total in-bore time. In this study, we chose parameters and range of values in the experiment protocol so they could be adapted to clinical PC-MRI measurements in small arteries in patients. Increased voxel size can reduce sequence time and increasing NSA will increase sequence time. If VENC is set to low, aliasing occurs and the sequence must either be repeated with corrected VENC to produce valid results, which will increase in-bore time, or the results adjusted with post-processing software.

### Study limitations

The main limitation when applying our results to a clinical setting is that our study was conducted with only constant flow. Limitations in temporal resolution in pulsatile flow will also affect the accuracy and precision of the measurements, affecting the systolic measurements more than the diastolic measurements as the systolic phase is shorter and more influenced by the temporal resolution. Another major limitation is that the use of a phantom fails to replicate the conditions *in vivo* (*e.g.*, signal characteristics of tissue, normal physiological variations). In our phantom, we used a plastic tube without an MRI signal, which probably leads to a larger overestimation of flow rate measurements compared to measurements done in the clinical setting. We used a fluid with low T1 to improve signal, but it is possible that if we had used a fluid with signal characteristics similar to blood, SNR would have a larger impact on our measurements. ROI selection is critical for accurate PC-MRI flow rate measurements, especially in small vessels where the proportion of boundary voxels is high. We chose to place the ROI based on the signal properties of images obtained in the baseline setting. We chose to examine three parameters that can influence the quality of measurements, but there are several other potential sources of error that were not considered in this study, for instance, deviation from the ideal imaging plane, flip angle, and bandwidth. We found a good precision in PC-MRI measurements, and as LDD in the standard reference and the baseline PC-MRI setting was similar, it is possible that some of the fluctuations seen in the different MRI settings are in fact true fluctuations in the flow rate and not effects of the precision of PC-MRI methods.

## Conclusions

Our results show that it is possible to measure flow rate and velocity with PC-MRI in a vessel phantom with a small lumen diameter with good precision but overestimation of flow rate measurements. Precision in flow rate measurements with PC-MRI at baseline setting was similar to our standard reference. With adjustments of the acquisition parameters, it is possible to achieve improvements in accuracy and/or precision.

## Data Availability

The datasets used and/or analyzed during the current study are available from the corresponding author on reasonable request.

## Abbreviations

CoV: Coefficient of variation
LDD: Least detectable difference
NSA: Number of signal averages
PC-MRI: Phase-contrast MRI
ROI: Region of interest
SD: Standard deviation
SNR: Signal-to-noise ratio
VENC: Velocity encoding
